# The Increase of Circulating PD-1- and PD-L1-Expressing Lymphocytes in Endometriosis: Correlation with Clinical and Laboratory Parameters

**DOI:** 10.1155/2018/7041342

**Published:** 2018-11-25

**Authors:** Monika Walankiewicz, Ewelina Grywalska, Grzegorz Polak, Izabela Korona-Glowniak, Elzbieta Witt, Agata Surdacka, Jan Kotarski, Jacek Rolinski

**Affiliations:** ^1^I Department of Gynecology, Medical University of Lublin, 16 Staszica Street, 20-081 Lublin, Poland; ^2^Department of Clinical Immunology and Immunotherapy, Medical University of Lublin, 4a Chodzki Street, 20-093 Lublin, Poland; ^3^Department of Pharmaceutical Microbiology with Laboratory for Microbiological Diagnostics, Medical University of Lublin, 1 Chodzki Street, 20-093 Lublin, Poland; ^4^Augusta Kliniken Bochum, 26 Bergstrasse Street, 44791 Bochum, Germany

## Abstract

The cause of endometriosis remains unknown. However, studies investigating the link between this condition and the immune system revealed several immunological abnormalities focused on cell-mediated immunity. As a major immune checkpoint, programmed cell death protein 1 (PD-1) displays an important inhibitory function in the maintenance of peripheral tolerance. The expression of PD-1 and its ligand (PD-L1) may contribute to continuous T cell activation and development of inflammation and injury of the tissue. To our knowledge, this is the first study evaluating frequencies of PD-1-positive T CD3^+^ cells (CD4^+^ and CD8^+^) and B cells (CD19^+^) in patients with endometriosis. Peripheral blood (PB) samples from 25 female patients and 20 healthy age and sex-matched subjects serving as controls were used in the study. Using flow cytometric analysis, we assessed the differences in the frequencies of PD-1-positive T and B lymphocytes in the study group and healthy individuals. Alteration of the PD-1/PD-L1 axis may contribute to the pathogenesis of endometriosis, as patients with advanced disease are characterized by higher frequencies of PD-1-positive T and B cells. Expression of PD-1 and PD-L1 on T and B cells could represent the hallmark of immune system reaction to chronic antigenic exposition in patients with endometriosis.

## 1. Introduction

Endometriosis is a common gynecological estrogen-dependent disorder characterized by the presence of endometrial tissue outside the uterine cavity, mainly in pelvic peritoneum and ovaries. Although it is a frequent cause of hospitalization in gynecological wards, its etiology to date is undetermined [1, 2]. Sourial et al. hypothesized that the first step of the establishment of endometrial tissue outside the uterine cavity may be ectopically placed stem cells of endometrial or hematopoietic origin or abnormal endometrial differentiation of a resident tissue stem cell [3]. Endometrial progenitor-type cells in a lesion's environment are involved in initiating further proliferation and maintaining the disease. A dysfunctional immune clearance process and a genetic predisposition may also contribute to the development of the disease. Common treatment for endometriosis usually depends on the modification of the ovarian steroid hormones. The target of this therapy are terminally differentiated ectopic endometriotic cells, while the stem cells that propagate the disease may not be affected [4, 5].

According to different authors, the endometriosis prevalence rate is up to 5–10% among the general female population [1, 2, 4] and even up to 15% of reproductive-age women [5]. The clinical manifestation may differ from a typical chronic pelvic pain, dyspareunia, and dysmenorrhea to asymptomatic form in 25%–50% of the cases [4, 6, 7]. There is a close link between infertility and endometriosis—25%–50% of women with infertility are simultaneously suffering from endometriosis, though 30–50% of women with diagnosed endometriosis are at the same time infertile [6, 8]. The presence of endometriosis is associated with a lower probability of pregnancy over 3 years—36%, comparing to women with unexplained fertility—55%, respectively [8, 9].

Based on research on the interdependence of endometriosis and disorders of the immune system, a number of abnormalities in the immune system in patients with endometriosis was postulated. The impaired function of T cells, B cells, and NK cells, the increased levels of various proinflammatory cytokines and angiogenetic factors such as IL-6, IL-1, and TNF-*α*, and the production of autoantibodies have been proved [10–13].

Programmed cell death 1 (PD-1) is a 288 amino acid encoded by the PDCD1 gene. This is an immunoreceptor belonging to the CD28/B7 superfamily of costimulatory molecules, which is involved in the regulation of the mechanisms of T cell tolerance. It is expressed on various cells, such as activated CD4^+^ T cells and CD8^+^ T cells, as well as antigen presenting cells [14–17]. PD-1 is composed of transmembrane receptor protein, an IgV-like extracellular domain, and a cytoplasmic part containing an immunoreceptor tyrosine-based inhibitory motif (ITIM) and an immunoreceptor tyrosine-based switch motif (ITSM) [18–21]. PD-1 may be expressed mainly on the surface of activated T cells, but they may also be expressed on the surface of several cells, such as B cells, NK cells, subsets of dendritic cells, and monocytes, and as a free molecule in the cytoplasm of naïve CD4^+^ T cells and the regulatory T cell Foxp3^+^ [22–25].

PD-1, together with its ligands programmed death ligand 1 (PD-L1) and PD-L2, plays an important role in inhibiting the maintenance of the immune homeostasis and peripheral tolerance. The binding of PD-1 to T cells and the secondary interaction with its ligand in combination with inhibited TCR signaling leads to negative costimulation and following T-cell anergy [26, 27]. Expression of PD-1 causes several changes; among others, it decreases the induction of cell survival protein Bcl-xL and expression of IFN-*γ* and IL-2. Blocking of the PD-1/PD-L1 pathway increases the response against antigen presented by DCs [28–31].

As the major immune checkpoint, PD-1 prevents autoimmunity, controls damage of the healthy tissues during infection, and promotes self-tolerance [20]. Interestingly, high PD-1 expression on the surface of T cells changes its ability to eliminate cancer and infectious disease [32–34]. The role of the PD-1/PD-L1 pathway in endometriosis is undetermined; however, the close relationship between endometriosis and the impaired immune response indicate that PD-1 may be involved in the pathogenesis of the disease.

## 2. The Aim of the Study

The aim of this study was to evaluate the frequencies of PD-1-positive and PD-L1-positive T CD3^+^ cells (CD4^+^ and CD8^+^) and B cells (CD 19^+^) in patients with newly diagnosed endometriosis in order to find a correlation between the increased expression of these molecules and the stage of the disease and clinical parameters.

## 3. Materials and Methods

### 3.1. Patients and the Control Group

The study group included 25 patients of the 1st Department of Oncological Gynecology and Gynecology of the Medical University of Lublin. Blood samples were taken from previously untreated women with newly diagnosed endometriosis two days before surgery. The mean age of the study group was 33.7 ± 6.7 years (median 29.5, Min–Max 18–49 years). The control group involved 20 healthy women with the mean age of 34.1 ± 7.0 years (median 35.5, Min–Max 19–44 years).

The characteristics of the study and the control groups are presented in Table 1.

The criteria for exclusion from the study were taking medications that affect the immune system, hormonal preparations, signs of infection occurring at least 4 weeks prior to the study, blood transfusion, and the occurrences of autoimmune disease, pregnancy, lactation, cancer, or allergies. The study protocol was submitted to the Institutional Review Board of the Medical University of Lublin and accepted (consent of Institutional Review Board of the Medical University of Lublin: KE-0254/302/2014) before the start of the study. All patients gave written informed consent to use their blood samples for scientific purposes.

Endometriosis was diagnosed based on standard diagnostic criteria and confirmed postoperatively. The stage of endometriosis was evaluated according to the revised American Society of Reproductive Medicine (rASRM) classification [35].

### 3.2. Material

Material for the study was 5 ml of peripheral blood drawn from the basilic vein of endometriosis patients and healthy individuals into lithium heparin-treated tubes (S-Monovette, SARSTEDT, Aktiengesellschaft & Co., D-51588 Nubrecht, Germany). Blood samples were immediately examined by flow cytometry at the Department of Clinical Immunology, the Medical University of Lublin.

#### 3.2.1. Isolation of Peripheral Blood Mononuclear Cells (PBMCs)

Isolation of PBMCs was conducted as described previously [31]. Peripheral blood samples with an amount of 5 ml were diluted in 0.9% buffered physiological saline—PBS without calcium (Ca^2+^) and magnesium (Mg^2+^) (Biochrom AG, Germany)—at a ratio of 1 : 1. The obtained material was initially transferred on 3 ml of Gradisol L preparation (Aqua Medica, Poland) with a specific gravity of 1.077 g/ml followed by density gradient centrifugation at an acceleration 700 ×g for 20 minutes. In this manner, two layers of the interphase were obtained. The PBMC fraction was collected with a Pasteur pipette and washed twice in PBS buffer without Ca^2+^ and Mg^2+^ for 5 minutes. Afterwards, the cells were suspended in 1 ml PBS buffer without Ca^2+^ and Mg^2+^, and counted in a Neubauer chamber. Their viability was assessed by trypan blue exclusion (0.4% Trypan Blue Solution, Sigma-Aldrich, Germany).

#### 3.2.2. Flow Cytometry of PD-1^+^ and PD-L1^+^ T CD4^+^, T CD8^+^, and B CD19^+^ Lymphocytes

Flow cytometric analysis of PD-1^+^ and PD-L1^+^ T CD4^+^, T CD8^+^, and B CD19^+^ lymphocytes was performed as described previously [31]. After PBMC isolation, the resulting cell suspension was distributed into individual tubes with an amount of 1 × 10^6^/sample, and the samples were incubated with a combination of the following mAbs: CD45 FITC/CD14 PE, Cy-Chrome Mouse Anti-Human CD3, FITC Mouse Anti-Human CD19, FITC Mouse Anti-Human CD4, FITC Mouse Anti-Human CD8, PE Mouse Anti-Human CD279 (PD-1), and PE Mouse Anti-Human CD274 (PD-L1), all of which came from BD Biosciences (USA). To the treated cells, 20 *μ*l of the antibody was added and all samples were incubated for 20 minutes at room temperature, washed twice with phosphate-buffered saline (PBS) (700 ×g, 5 min), and immediately analyzed in a FACSCalibur flow cytometer (BD Biosciences, USA). Data acquisition was performed using the specialized FACSDiva Software 6.1.3 (BD Biosciences, USA) with 30,000 cells analyzed during each run. The results of flow cytometric analysis were presented as percentage of stained cells with monoclonal antibodies conjugated with fluorescent dyes and as a mean fluorescence intensity (MFI), which is the exponent of the expression values of a given cell surface antigen.

Background fluorescence was assessed using isotype-matched directly conjugated FITC Mouse IgG1 *κ* Isotype Control and PE Mouse IgG1 *κ* Isotype Control monoclonal antibodies. To exclude debris and cell aggregates, the samples were gated on forward scatter vs. side scatter.

**Figure 1:**
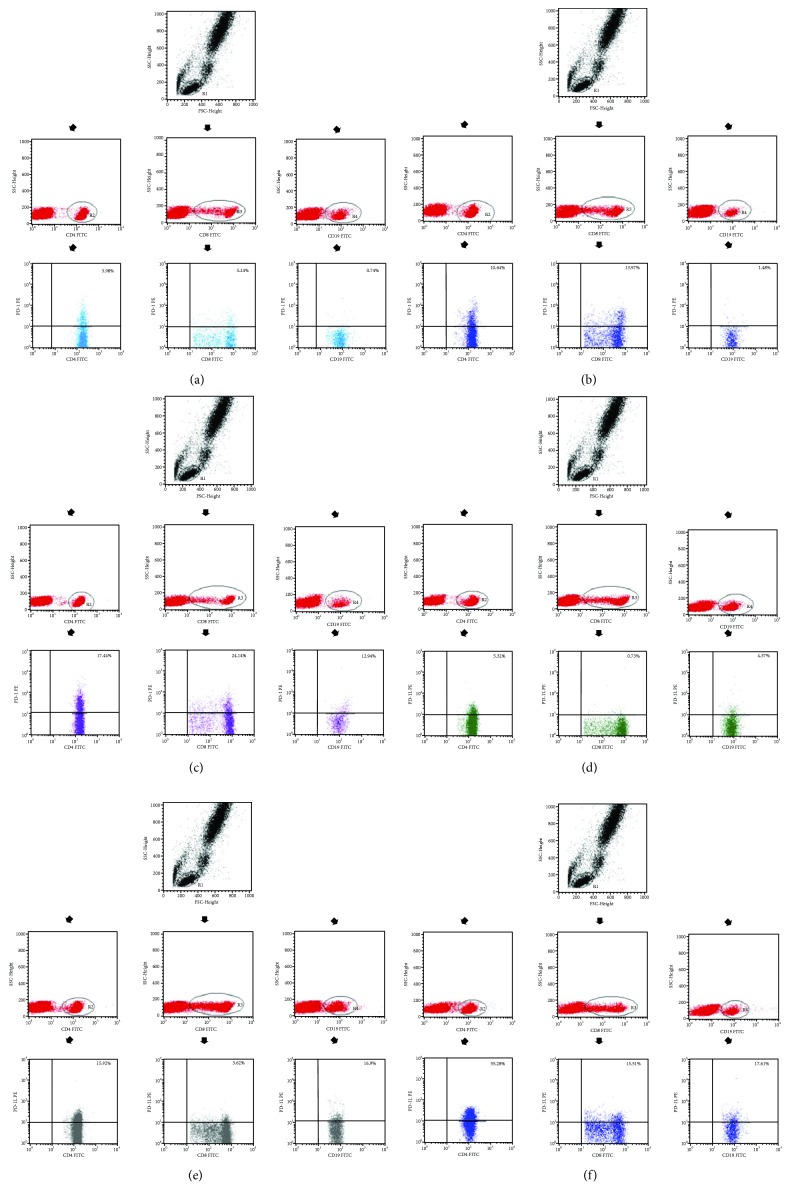
(a) Sample analysis of the expression of PD-1^+^ on CD4^+^ T, CD8^+^ T, and CD19^+^ B lymphocytes in a healthy individual. (b) Sample analysis of the expression of PD-1^+^ in CD4^+^ T, CD8^+^ T, and CD19^+^ B lymphocytes in a stage I endometriosis patient. (c) Sample analysis of the expression of PD-1^+^ in CD4^+^ T, CD8^+^ T, and CD19^+^ B lymphocytes in a stage IV endometriosis patient. (d) Sample analysis of the expression of PD-L1^+^ in CD4^+^ T, CD8^+^ T, and CD19^+^ B lymphocytes in a healthy individual. (e) Sample analysis of the expression of PD-L1^+^ in CD4^+^ T, CD8^+^ T, and CD19^+^ B lymphocytes in a stage I endometriosis patient. (f) Sample analysis of the expression of PD-L1^+^ in CD4^+^ T, CD8^+^ T, and CD19^+^ B lymphocytes in a stage IV endometriosis patient.

An example of flow cytometry analysis is shown in Figure 1.

### 3.3. Statistical Analysis

Statistical analysis was carried out using appropriate statistical tests and Statistica v. 10.0PL software (StatSoft, USA). The values of the parameters were presented as arithmetic means and standard deviations (SD), medians, minimum and maximum value, upper and lower quartiles, and the range of variability. Shapiro-Wilk test was applied to test normal distribution of continuous variables. The Student *t*-test was used for independent variables, and the Mann–Whitney *U* test was used as the intergroup comparison component. Kruskal-Wallis ANOVA and multiple comparisons of mean ranks (as post hoc analysis) were applied for the analysis of differences between more than two groups. The distribution of discrete variables in groups was compared with the Pearson's Chi-square test or the Fisher's exact test. A *p* value < 0.05 was considered statistically significant.

## 4. Results

### 4.1. Frequencies of PD-1-Positive and PD-L1-Positive T and B Lymphocytes in Endometriosis Patients and the Control Group

**Figure 2:**
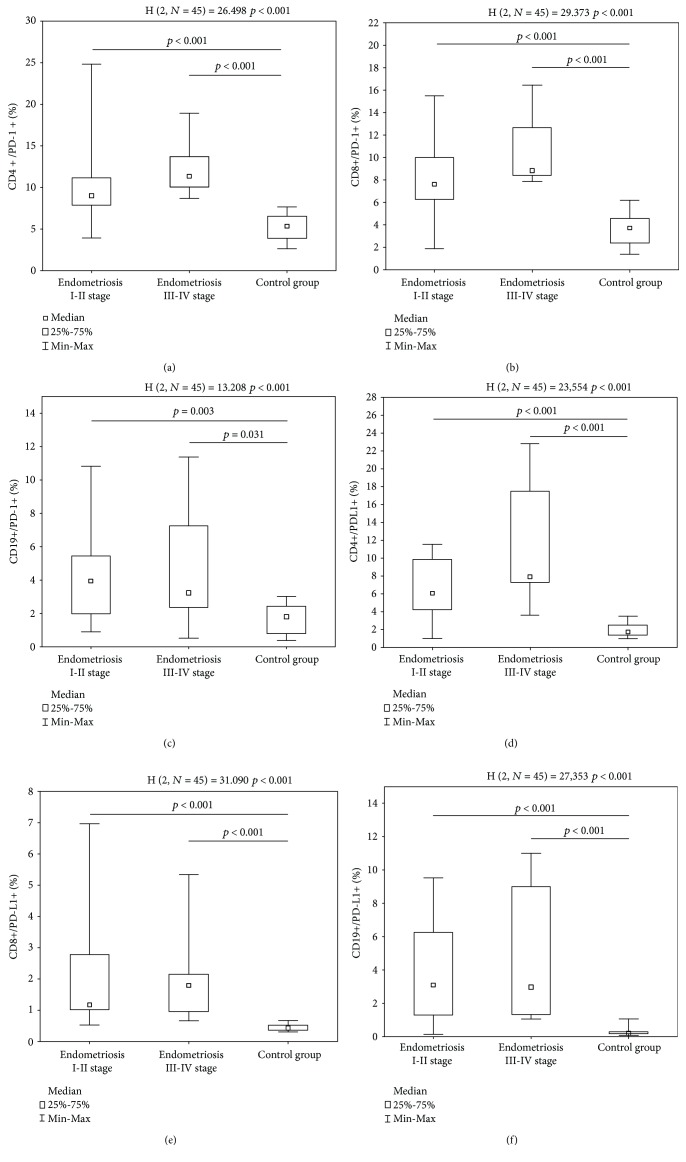
(a) The frequencies of CD4^+^/PD-1^+^ cells in stage I and II endometriosis patients, stage III and IV endometriosis patients, and healthy volunteers. (b) The frequencies of CD8^+^/PD-1^+^ cells in stage I and II endometriosis patients, stage III and IV endometriosis patients, and healthy volunteers. (c) The frequencies of CD19^+^/PD-1^+^ cells in stage I and II endometriosis patients, stage III and IV endometriosis patients, and healthy volunteers. (d) The frequencies of CD4^+^/PD-L1^+^ cells in stage I and II endometriosis patients, stage III and IV endometriosis patients, and healthy volunteers. (e) The frequencies of CD8^+^/PD-L1^+^ cells in stage I and II endometriosis patients, stage III and IV endometriosis patients, and healthy volunteers. (f) The frequencies of CD19^+^/PD-L1^+^ cells in stage I and II endometriosis patients, stage III and IV endometriosis patients, and healthy volunteers.

Using the flow cytometric analysis, the differences in the frequencies of PD-1-positive and PD-L1-positive T lymphocytes and B lymphocytes were assessed in the study group and healthy individuals. The study group was divided into two subdivisions, i.e., endometriosis stages I and II and advanced endometriosis (stages III and IV). CD4^+^/PD-1^+^ T cells were present more frequently among patients suffering from endometriosis stages I and II (9.01 ± 4.75%, median 9.01%, Min–Max 3.92–24.84%) and stages III and IV (12.36 ± 3.37%, median 11.33%, Min–Max 8.67–18.92%), than among patients from the control group (5.35 ± 1.54%, median 5.35%, Min–Max 2.65–7.69%) (Figure 2(a)). The frequencies of CD8^+^/PD-1^+^ T cells in patients with endometriosis stages I and II (8.19 ± 3.76%, median 7.59%, Min–Max 1.90–15.48%) and III and IV (10.40 ± 3.11%, median 8.81%, Min–Max 7.83–16.45%) exceeded those of the healthy subjects (3.60 ± 1.46%, median 3.71%, Min–Max 1.36–6.17%) (Figure 2(b)). The frequencies of CD19^+^/PD-1^+^ B cells were also higher in patients with endometriosis stages I and II (4.07 ± 2.59%, median 3.95%, Min–Max 0.9–10.82%) and stages III and IV (4.56 ± 3.63%, median 3.23%, Min–Max 0.54–11.37%) than in those of healthy subjects (1.67 ± 0.84%, median 1.81%, Min–Max 0.37–3.01%) (Figure 2(c)).

Likewise, the CD4^+^/PD-L1^+^ T cells were present more frequently in patients with endometriosis stages I and II (6.56 ± 3.44%, median 6.06%, Min–Max 0.97–11.51%) and stages III and IV (11.00 ± 6.72%, median 7.89%, Min–Max 3.58–22.79%) than in those from the control group (1.86 ± 0.70%, median 1.71%, Min–Max 0.98–3.49%) (Figure 2(d)). Correspondingly, patients from the stage I and II endometriosis group (1.98 ± 1.67%, median 1.17%, Min–Max 0.52–6.97%) and stage III and IV endometriosis group (2.06 ± 1.55%, median 1.79%, Min–Max 0.66–5.34%) had higher frequencies of CD8^+^/PD-L1^+^ T cells than healthy individuals (0.45 ± 0.11%, median 0.43%, Min–Max 0.31–0.67%) (Figure 2(e)). Finally, the frequencies of CD19^+^/PD-L1^+^ B cells were higher in patients with stage I and II endometriosis (3.85 ± 3.08%, median 3.08%, Min–Max 0.13–9.51%) and in patients with more advanced stage III and IV endometriosis (4.57 ± 3.88%, median 2.97%, Min–Max 1.05–10.98%) than in patients from the control group (0.26 ± 0.23%, median 0.20%, Min–Max 0.07–1.03%) (Figure 2(f)).

The same pattern was observed, when looking at the proportions between the expression of the PD-1 receptor and PD-L1 on the examined cells. The CD4^+^/PD-1 : CD4^+^/PD-L1^+^ T cell ratio was lower in the endometriosis group (1.84 ± 1.23, median 1.45, Min–Max 0.57–6.26) than in the healthy subjects (3.1 ± 1.2, median 2.83, Min–Max 3.1 ± 1.2) (*p* < 0.0001). The CD8^+^/PD-1 : CD8^+^/PD-L1^+^ T cell ratio was also lower in the endometriosis group (6.36 ± 4.84, median 5.57, Min–Max 1.47–24.17) than in the healthy subjects (8.37 ± 4.01, median 7.42, Min–Max 3.58 ± 17.63) (*p* = 0.031). Finally, the CD19^+^/PD-1 : CD19^+^/PD-L1^+^ B cell ratio was significantly lower in patients (1.74 ± 1.66, median 1.23, Min–Max 0.2–7.77) than in the control group (9.4 ± 7.8, median 7.3, Min–Max 1.5–27.4) (*p* < 0.0001). There was no statistical difference between endometriosis stages I and II and the more advanced stages III and IV (*p* = 0.31).

**Figure 3:**
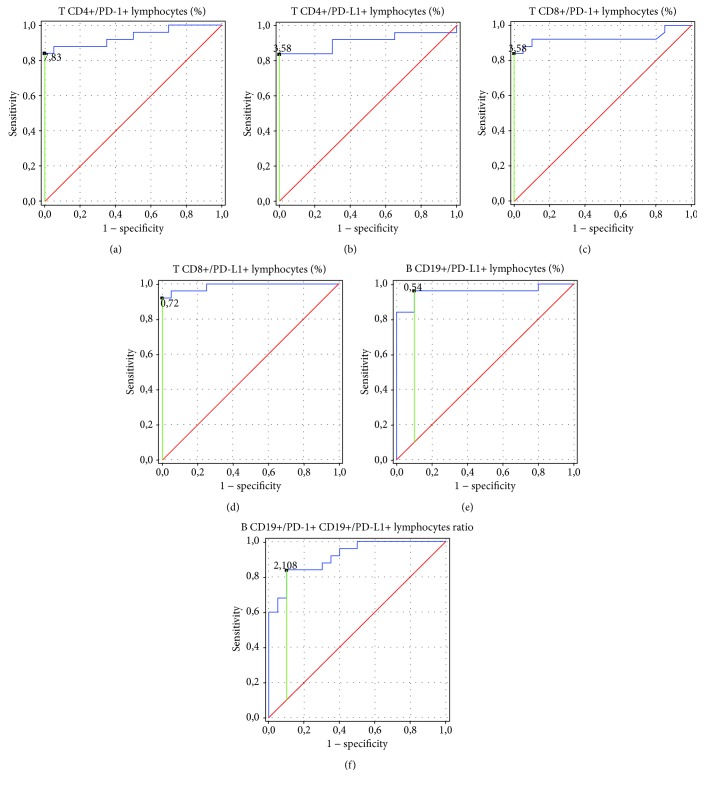
Receiver operating characteristic (ROC) curve to compare immunological parameter sensitivity and specificity in patients with endometriosis. (a) ROC curves of CD4^+^/PD-1^+^lymphocytes (%). (b) ROC curves of CD4^+^/PD-L1^+^lymphocytes (%). (c) ROC curves of CD8^+^/PD-1^+^ lymphocytes (%). (d) ROC curves of CD8^+^/PD-L1^+^ lymphocytes (%). (e) ROC curves of CD19^+^/PD-L1^+^ lymphocytes (%). (f) ROC curves of CD19^+^/PD-1^+^ lymphocytes: CD19^+^/PD-L1^+^ lymphocyte ratio.

Figure 3 and Table 2 show the ROC analysis of the nine immunological parameters related to PD-1 and PD-L1 biomarkers. As the areas under the curve (AUC) have revealed it, the frequencies of CD8^+^/PD-L1^+^ T lymphocytes were the most sensitive and specific parameter to determine in patients with endometriosis (AUC = 0.988). Diagnostic accuracy was excellent for increasing the parameters of the frequencies of CD4^+^/PD-1^+^ T lymphocytes and CD8^+^/PD-1 T lymphocytes. The parameters of PD-L1 presented on CD4^+^ T lymphocytes and CD19^+^ B lymphocytes showed excellent diagnostic accuracy as well. The CD19^+^/PD-1^+^ : CD19^+^/PD-L1 B lymphocyte ratio below their prognostic value was perfect for discriminating between endometriosis and nondiseased patients.

### 4.2. Comparison between Laboratory Parameters in Patients with Different Stages of Endometriosis

There were no statistical differences of blood count parameters and Ca-125 level between stage I and II and stage III and IV endometriosis patients. HE4 was the only parameter statistically different in stages I and II (38.83 ± 4.69 pmol/l, median 39.4 pmol/l, Min–Max 28.5–48.0 pmol/l) compared to stages III and IV (60.07 ± 4.41 pmol/l, median 60.5 pmol/l, Min–Max 53.5–65.0 pmol/l) (*p* = 0.0002).

### 4.3. Comparison between Laboratory Parameters in Patients with Different Manifestations of Endometriosis

We observed a higher CD8^+^/PD-1 : CD8^+^/PD-L1^+^ T cell ratio in patients with dysmenorrhea (7.35 ± 5.14, median 6.76, Min–Max 1.5–24.2) compared with other endometriosis patients (3.24 ± 1.46, median 2.99, Min–Max 1.5–5.6) (*p* = 0.028).

Endometriosis patients with adhesions presented statistically lower frequencies of CD8^+^/PD-L1^+^ T cells (1.35 ± 0.83, median 1.05%, Min–Max 0.5–3.6%) than in those from the other endometriosis groups (2.72 ± 1.95, median 2.13%, Min–Max 0.9–6.97%) (*p* = 0.036). Similarly, we observed a higher CD8^+^/PD-1 : CD8^+^/PD-L1^+^ T cell ratio (8.11 ± 5.71, median 6.95, Min–Max 1.9–24.2) compared with other endometriosis patients (4.46 ± 2.84, median 4.09, Min–Max 1.5–10.6) (*p* = 0.047).

## 5. Discussion

PD-1 and its ligands, PD-L1 and PD-L2, play an important role in inhibitory signaling pathways that regulate T cell response and maintain peripheral tolerance. The PD-1/PD-L1 pathway plays a critical role in regulating autoreactive lymphocytes and providing balance between protective immunity and tolerance [25, 36]. It was shown that PD-1 inhibits the proliferation of naïve and effector T cells and the production of several cytokines; it also influences autoreactive T cells [37, 38]. The complexity and multifactorial etiology of endometriosis indicates the role of negative costimulation in its development.

Our study revealed increased frequencies and numbers of PD-1-positive and PD-L1-positive CD3^+^CD4^+^ and CD3^+^CD8^+^ T cells, as well as CD19^+^ B cells in endometriosis patients. At the same time, PD-L1 and PD-1 expressions were observed to increase with the advance of the disease. These findings were significant in patients with endometriosis, and not observed in the control group. The PD-1/PD-L1 pathway is one of the main negative costimulatory signaling pathways taking part in maintaining the peripheral tolerance and regulation of autoreactive lymphocytes. The role of PD-1 was described in the pathogenesis of infectious and autoimmune diseases and many types of cancer as well as transplantology. To date, this is the first study showing correlations between endometriosis and the PD-1/PD-L1 pathway.

The first investigation was conducted on PD-1-deficient animals by Nishimura et al. The exposition of C57BL/6(B6)-PD-1^−^ transgenic mice to Fas mutation results in spontaneously developed lupus-like proliferative arthritis and glomerulonephritis with predominant IgG3 deposition as well as dilated cardiomyopathy with the presence of cardiac troponin I antibodies. Collectively, PD-1 deficient 2C-TCR (anti-H-2L^d^) transgenic mice developed chronic and systemic graft-versus-host-like disease. The investigation of CD8^+^2C-TCR^+^PD-1^−/−^ T cells revealed that their proliferation in vitro was remarkably augmented in response to H-2^d^ allogeneic cells. All the abovementioned data indicate the role of PD-1 in the maintenance of peripheral self-tolerance by a negative regulation of immune responses [39, 40].

Dmowski et al. suggest that development of endometriosis results from a congenital or acquired defect of the immune system [41]. Concomitantly, with an immune dysfunction, women with endometriosis also are more likely to develop autoimmune diseases like systemic lupus erythematosus, Sjögren syndrome, rheumatoid arthritis, multiple sclerosis, and allergies. The link between endometriosis and systemic lupus erythematosus (SLE) was indicated by Pasoto et al. who described the presence of similar symptoms in endometriosis and systemic lupus erythematosus (SLE). However, none of the endometriosis patients in the study was diagnosed with SLE according to the minimum criteria for SLE. Endometriosis patients in the study were reported to have a significantly higher incidence of arthralgia (62%) and myalgia (18%) compared with normal controls, but the incidence rate was similar to that reported by SLE patients [42, 43]. It also allows us to suppose that there is a common source of these immunological disorders.

It was proven that the alteration of the PD-1/PD-L1 axis is involved in the pathogenesis of the abovementioned autoimmune disorders. The PD-1 gene presence is significantly associated with rheumatoid arthritis susceptibility, showing the possibility that PD-1 may be involved in the pathogenesis of the disease [44, 45]. The following studies show that the course of the disease is related to an additional soluble PD-1 splicing variant blocking PD-1 [46]. Using the collagen-induced arthritis model, Wang et al. proved the reduction of the expression of proinflammatory cytokines by administering recombinant PDL-Ig [45]. Kobayashi et al. indicated a dysfunction of the PD-1/PD-L1 pathway in patients with Sjögren syndrome, showing that PD-1 is expressed in T lymphocytes and PD-L1 in epithelial cells of inflamed salivary glands [47]. It was proven that PD-1 deficiency influences the development of autoimmune diseases due to the lack of inhibition of activated lymphocyte proliferation. Therefore, it may be assumed that these cells may escape the immune system [48].

The correlation between PD-1 and infertility was under investigation. Zamani et al. considered PD-1 gene polymorphism at the level of single nucleotide polymorphisms (SNP) of the genome and susceptibility to antisperm antibody-related infertility in an Iranian group of infertile patients. It was assessed whether their association with prognostic factors had a correlation between impaired PD-1-associated immunomodulation and development of antisperm antibodies [49].

It is worth considering PD-1 blockade as an important treatment tool for endometriosis patients. So far, PD-1/PD-L1 pathway blockade has been studied in several gynecological malignancies, including epithelial ovarian cancer (EOC) and endometrial cancer. Durable and significant responses were observed when patients with recurrent EOC were treated with checkpoint inhibitors; however, only a proportion of patients will respond to checkpoint inhibitor monotherapy. These results are interesting and need follow-up studies [50–52]. Pembrolizumab, an anti-PD-1 inhibitor, turned out to be effective in the treatment of advanced endometrial cancer. Preliminary results from the phase Ib KEYNOTE-028 (National Clinical Trial (NCT02054806)) of 24 patients with highly advanced endometrial cancer also suggest an activity for pembrolizumab with tolerable toxicity. At a median of 69.9 weeks of follow-up, confirmed overall response rate was 13% (3/24) and another 13% (3/24) achieved stable disease [51, 53]. An ongoing phase II study of pembrolizumab has shown an overall response rate of 55.6% (5/9) and a clinical benefit rate of 88.9%. Of interest was one patient who achieved a sustained complete response (CR) for 17 months and had previously progressed through 3 prior lines of chemotherapy [51, 54].

It was suggested that endometriosis-like lesion growth and development are affected by estrogen receptors (ER) depending on estradiol-mediated activity [55]. Elevated ER*β* expression was observed in endometriotic tissue and stromal cells. Levels of ER*β* were 36 times higher than those in normal endometrium or stromal cells, suggesting that the biological effect of estrogen on endometriosis is mediated at least partially through ER*β* [56, 57]. Recently, many studies have focused on the relationship of the PD-1/PD-1L pathway and sex hormones. The first such study, conducted by Polanczyk et al., demonstrated that treating EAE (experimental autoimmune encephalomyelitis) mice with 17*β*-estradiol (E2) leads to increased CD4^+^CD25^+^ Treg compartment, FoxP3 expression, and expression of PD-1 on Tregs, macrophages, B cells, and dendritic cells. This implicates the role of estradiol in the upregulation of PD-1 and suggests that PD-1 ligation may act as a positive or a negative costimulatory pathway in T-cell activation, depending on the ratio of T-cells and dendritic cells [58]. Wang et al. indicate that intracellular expression of PD-1 Treg cell is a critical mediator of estradiol-induced protection against EAE [59].

## 6. Study limitations

We did not evaluate patients' PD-1^+^ and PD-L1^+^ lymphocytes after surgery. It was impossible as all the patients were treated postoperatively with analgesics, progestins (oral/depot/interuterine progestin-releasing system), combined estrogen and progestin therapy, gonadotropin-releasing hormone agonist, and danazol or aromatase inhibitors, and all the abovementioned medications affect the immune system [60, 61]. For the same reason, we could not assess whether a correlation existed between the frequencies of PD-1^+^ and PD-L1^+^ lymphocytes after surgery and patients' fertility outcome. The assessment of association between PD-1 and PD-L1 expression and endometriosis recurrence was also impossible. Our preliminary data are very interesting/promising but they require confirmation and extending by describing the PD-1/PD-L1 pathway in patients untreated postoperatively with medications that affect the immune system.

## 7. Conclusions

This study showed that the percentage and number of PD-1 and PD-L1-positive CD3^+^CD4^+^ T cells, CD3^+^CD8^+^ T cells, and CD19^+^ B cells are significantly higher in the study group than in the control group. Reduced PD-1 expression in endometriosis is also observed in several autoimmune diseases, which indicates some similarities in the pathogenesis of both diseases. Additionally, according to the findings of our study, the deregulation of the PD-1/PD-L1 pathway may be associated with poorer prognosis, since patients with moderate and severe endometriosis are characterized by higher frequencies of PD-1-positive T and B cells. PD-1 and PD-L1 expression may contribute to continuous T cell activation and development of inflammation and injury of the tissue. Further investigation is needed to consider the role of the blockade of the PD-1/PD-L1 axis and its influence on disease progression. Similarly, further research regarding the place of PD and PD-L1 in endometriosis is needed. For example, it is important to relate the expression of PD-1 and PD-L1 on lymphocytes to their expression in endometrial lesions, which will require histological and immunohistochemical analyses.

## Figures and Tables

**Table 1:** Characteristics of the study and the control groups.

Parameter	Endometriosis (*n* = 25)	The control group (*n* = 20)	*P* value
Age (years) mean ± SD	33.7 ± 6.7	34.1 ± 7.0	NS
Stage of the disease:			
I and II	72% (*n* = 18)	N/A	—
III and IV	28% (*n* = 7)		
Adhesions	52% (*n* = 13)	N/A	—
Dysmenorrhea	76% (*n* = 19)	N/A	—
Infertility	32% (*n* = 8)	N/A	—
Haemoglobin (g/dl) mean ± SD	13.3 ± 0.9	13.4 ± 1.1	NS
Leukocytosis (×10^3^ cells/*μ*l) mean ± SD	7.8 ± 0.9	6.8 ± 0.4	NS
Platelets (×10^3^ cells/*μ*l) mean ± SD	253 ± 53.1	279 ± 57.1	NS
Ca-125 (U/ml) mean ± SD	44.8 ± 10.7	9.23 ± 5.4	*p* < 0.0001
HE-4 (pmol/l) mean ± SD	33.7 ± 6.7	37.1 ± 9.1	NS

NS = not significant, N/A = not applicable.

**Table 2:** ROC analysis to determine diagnostic accuracy of immunological parameters in endometriosis patients.

Parameter	Prognostic value	AUC	95% CI
CD4^+^/PD-1^+^ lymphocytes (%)	7.83	0.936	0.86–1.0
CD4^+^/PD-L1^+^ lymphocytes (%)	3.58	0.91	0.81–1.0
CD8^+^/PD-1^+^ lymphocytes (%)	6.22	0.927	0.84–1.0
CD8^+^/PD-L1^+^ lymphocytes (%)	0.72	0.988	0.97–1.0
CD19^+^/PD-L1^+^ lymphocytes (%)	0.54	0.956	0.89–1.0
CD19^+^/PD-1^+^ : CD19^+^/PD-L1^+^ lymphocyte ratio	2.11	0.918	0.84–0.99

## Data Availability

The datasets analyzed during the current study are available from the corresponding author on reasonable request.
